# Supplementary material for: Hunting strategies to increase detection of chronic wasting disease in cervids
Source: Nat Commun. 2020 Sep 1;11:4392. doi: 10.1038/s41467-020-18229-7 (PMC7463264; doi:10.1038/s41467-020-18229-7)
Supplement: Supplementary file 1 — Supplementary Information [file 41467_2020_18229_MOESM1_ESM.pdf]

Supplementary Information

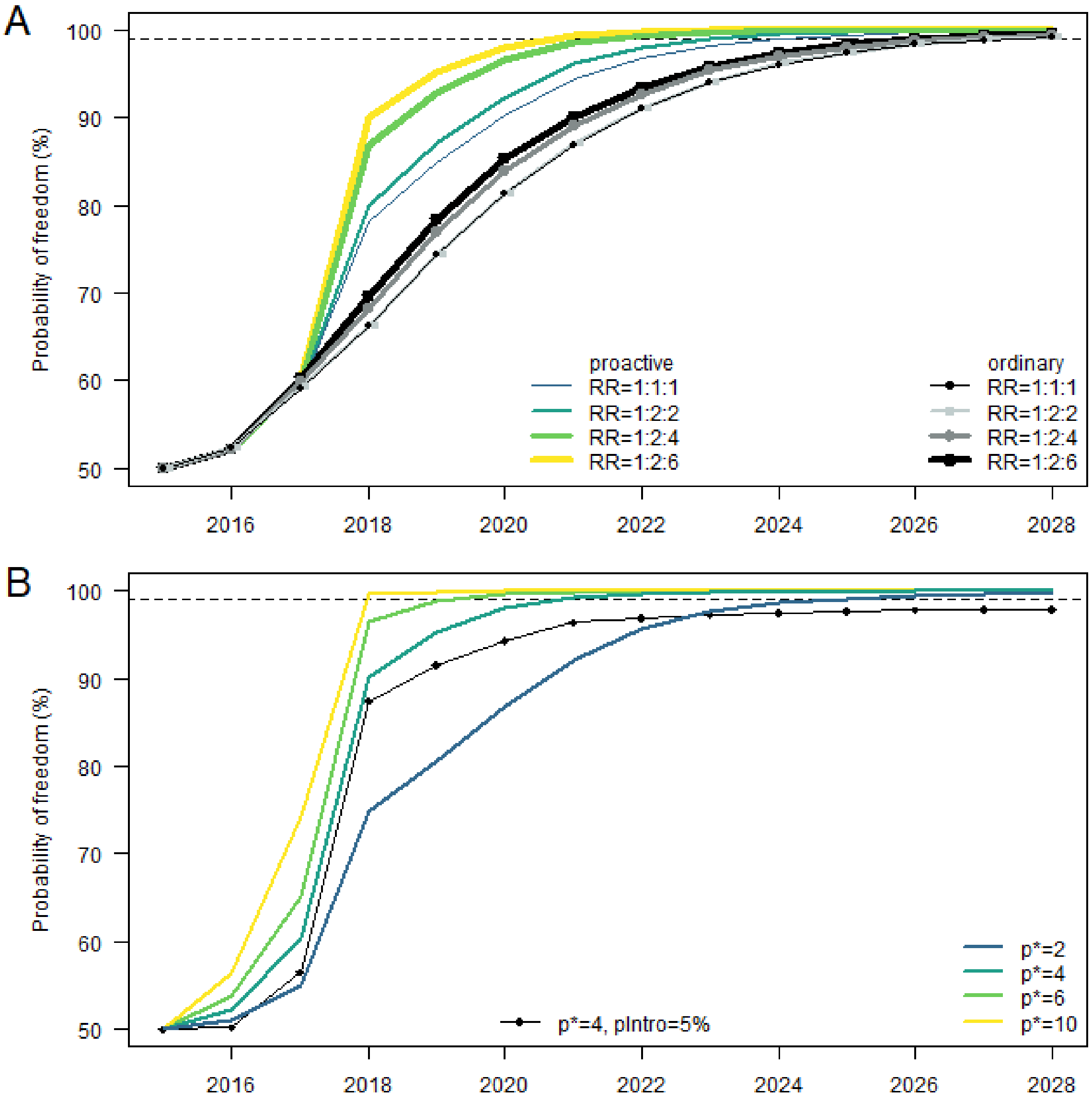

Supplementary Figure 1. (A) The effect of varying the relative risk (RR) of infection among age and sex classes for the Hardangervidda reindeer population, Norway. The effect of the RR is compared between a strategy of culling only adults and also culling available adult males to reach a sex ratio (m:f) of 1:5 and the strategy of surveillance for the ordinary harvest. (B) The effect of epidemiological uncertainty on the time to reach a given probability of freedom from infection. Design prevalence (p*) is the number of infected individuals to be detected, which will depend on the assumed time of infection, rate of transmission and assessed importance of early detection. Risk of infection introduction (pIntro) were set to 1% and 0.1%, corresponding to before and after the adjacent infected population was culled, and compared to a scenario of keeping a high risk of introduction (5%) throughout all years.

Supplementary Table 1 An overview of parameters for the different model compartments used in a model to estimate the probability of reaching freedom from infection, as exemplified by two reindeer populations that were potentially infected by CWD.

**Parameter**	**Notation**	**Value**	**Principle and basis**	**Comments and limitations**
**Population model**				
Initial population size, demographic structure, survival and reproductive rates		[initial population size 2018]<br>Nordfjella zone 2: [650]<br>Hardangervidda: [8300]	Empirical. Informed and estimated by annual winter, summer and fall surveys, and harvest data	Previously published model structure[1], fitted to new data from years 2015-2018 (Nordfjella) and 2000-2018 (Hardangervidda).
**Population part of simulations**				
Operational sex ratio	SR (m:f)	[1:3, 1:10, 1:20]	Population management decision guided by empirical knowledge. In polygynous species, one male can inseminate many females	An excessively strong sex ratio skew may have adverse effects even in polygynous species, including by delaying calving, and reducing pregnancy, depending on the mating system[2]
Minimum female population	T_adf	Nordfjella zone 2: [150]<br>Hardangervidda: [3000]	Population management decision guided by theory to retain a sustainable population size	Knowledge regarding the minimum viable population size may guide choice[3]

Carrying capacity	K	Nordfjella zone 2: [800]<br><br>Hardangervidda: [10000]	Empirical. Keep population from exceeding carrying capacity.	Often difficult to determine carrying capacity empirically. For reindeer, avoiding overgrazing of lichen during the winter is essential[4]
Harvest rate and composition	h_{adm}, h_{adf}, h_{ym}, h_{yf}, h_{cm}, h_{cf}	To be optimized depending on management goal, h_{adf} varies relative to closeness of population to K. Function scaled (h_{adf} = hscale – hscale*(K-N_{total})/K) to ensure that for the first year of simulated data, h_{adf}, is close to the three-year average of estimated harvest rates (2016-2018). Set as the proportion of a given age/sex class in the population (rather than as % of total population)	Output from the model and dependent on harvest strategy: average historical harvest rates or based on operational sex ratio	May be considerable implementation uncertainty in estimated levels (number of animals corresponding to a given harvest rate is dependent on the initial population size, population structure and demographic rates, and how to set a quota to obtain the suggested harvest).
**Disease detection model**				Previously published scenario tree-type of model[5], extended here with the demographic pattern of infection

Sample type, quality and test regime		Percentage of brain tissue and retropharyngeal lymph node versus brain tissue only. Nordfjella zone 2 (2018): 97.5%<br><br>Hardangervidda (2018): 74%	Partly empirical. Total sample size and proportion in each category. For coming years, the proportion from the last recorded year was used as input. Sample quality modelled with uncertainty.	In scenario-tree models, a crucial part of the data input is the number of tested units (for each category of a risk factor)[5]
Test sensitivity		Sensitivity of ELISA assumed to increase over disease course	Partly empirical. Modelled according to the stage of infection, known for the later stage of disease[6].	Fig. 1 in Viljugrein et al. (2019)[5]
Number of infected individuals in different infection stages		2 years duration from infection to death.	Partly empirical. Number of infected individuals at different stages depends on the incubation period. We assume that an infected individual has an equal probability of being anywhere on the time axis (time since infection to death). Level of disease-related mortality and rate of new infections may affect this relationship but is not incorporated here.	Limited knowledge of the CWD incubation period for reindeer[7,8]; more for white-tailed deer, mule deer [9] and elk[10]. Variation linked to genetics and prion strains affect incubation period.

Demographic pattern of infection and relative risk	RR	No pattern-for theoretical comparison:<br><br>Yearlings: 1<br><br>Adult females: 1<br><br>Adult males: 1<br><br>Adjusted for age categories:<br><br>Yearlings: 1<br><br>Adult females: 2<br><br>Adult males: 2<br><br>Adjusted for age categories and sex in adults:<br><br>Yearlings: 1<br><br>Adult females: 2<br><br>Adult males: 4 and 6	Empirical. Relative risk of infection differs among age and sex classes. Adjusted risk of infection is calculated according to demographic pattern of infection as adjusted by the demographic structure of the population. Relative risks are adjusted to ensure that the average relative risk for a representative sample of the reference population is 1, while maintaining the relative ratios as specified[11]	Some knowledge of the demographic pattern of CWD infection from reindeer[12], good data from white-tailed deer and mule deer[13,14,14,15], less from elk[16,17].<br><br>For sensitivity analysis, we also included RR as a stochastic betapert-distribution (bp) specified by minimum, maximum and mode value.<br><br>Adult females relative to yearlings: bp(1, 1.5 ,2.5) or bp(1, 2, 2.5)<br><br>and adult males relative to yearlings: bp(2, 4.5, 6.5) or bp(2, 6, 6.5).

**Epidemiological part of simulations**				This paper
Design prevalence (the prevalence of infection to be detected in the population)	p*	[Numbers are individuals]<br>Nordfjella zone 2: [2 in 2016, increasing to 3 from 2018, increasing to 4 from 2020]<br>Hardangervidda: [4]	Empirical in theory. In practice, expert judgement regarding expected epidemiological development (R0 ~ 2) and importance of early detection. In our case, set by Norwegian Food Safety Authority.	No empirical information to guide choice for CWD in reindeer, using epidemiological knowledge from mule deer[18,19].<br>Design prevalence was calculated relative to the population of adults and yearlings.
Probability of introduction	pIntro	Theoretical exploration<br>0.1%, 0.5%, 1% and 5%<br>To the CWD situation<br>Nordfjella zone 2: [5 %, 0.1 % from 2018]<br>Hardangervidda: [1 %, 0.1 % from 2018]	Empirical in theory. In practice, expert judgement regarding potential contact (historical and future) between population and Nordfjella zone 1. In our case, preliminary set by Norw. Vet. Inst.	Some knowledge about the likelihood of landscape barrier crossing by reindeer based on traditional knowledge and recent modelling using step-selection functions[20].
**Output variable**				

Surveillance sensitivity	SSe	Estimate at specified design prevalence	Estimation. The probability of detecting the disease from the specific sampling regime and at the specified design prevalence	The probability of detecting the disease from the specific sampling regime and at the specified design prevalence[5]
Freedom from infection	ProbFree	Aim: 99% probability within 5 years and reach 90% within first few years	Determining the certainty level and time to obtain the level is a management risk decision	Determining rapid freedom from infection requires large samples skewing the age and sex structure of population (development of this paper)

Supplementary Table 2. Parameter values and initial population size and structure used for the two areas (estimates from the Bayesian population model). Harvest rate is number harvested/mean population estimate of demographic group. In simulations we use the 3-yrs mean (2016-2018). c = calves; y = yearlings; ad=adults; m = males; f = females.

Parameter	Nordfjella zone 2	Hardangervidda
**Harvest rates** (h) as 3-yrs mean (range)		
h_{cf}	7.3% (3-15)	13.0% (3-22)
h_{cm}	7.7% (4-11)	15.3% (6-23)
h_{yf}	3.0% (2-5)	10.7% (3-19)
h_{ym}	13.7% (13-15)	21.3% (6-40)
h_{adf}	11.3% (8-14)	14.3% (4-25)
h_{adm}	14.3% (12-18)	18.0% (15-21)
**Demographic rates**[1] as mean (SD)		
Fertility rate	0.632 (0.043)	0.649 (0.027)
Summer surv. calves	0.948 (0.039)	0.942 (0.032)
Winter survival	0.971 (0.011)	0.934 (0.003)
**Population size 2018** (N) as mean (SD)		
N_{cf}	58 (4.8)	796 (62)
N_{cm}	80 (4.4)	759 (57)
N_{yf}	58 (4.5)	614 (30)
N_{ym}	64 (4.5)	562 (29)
N_{adf}	223 (4.7)	3113 (43)
N_{adm}	159 (5.5)	2427 (39)

[1] Mean demographic rates for Hardangervidda were estimated from a period of population decline (2008-2018)

Supplementary Table 3. An overview of different harvest scenarios (strategy) for Nordfjella zone 2, Norway. Sex ratios are ordinary (Suppl. Table 1), increased harvest to twice the rate of ordinary (ordinary*2) or given with male:female ratios. Hrate = harvest rate (h1 = Suppl. Table 1; h0 = no calves or yearling harvest). RR = relative risk (RR of yearlings, adult females and adult males relative to yearlings). pintro= probability of infection introduction in % (baseline 5% first year, 0.1% after). ProbFree = mean probability for freedom of infection. NR = not reached. A sensitivity analysis of pintro and RR is included for two harvest strategies (No1 and No6). A few scenarios run with stochastic RR were also included. Stochastic RR were assumed betapert distributed (βp) with minimum RR=1:1:2, maximum RR=1:2.5:6:5 and mode RR=1:2:6 or 1:1.5:4.5).

					After 1 year (2018)			After 5 years (2022)			Years to reach a given level of freedom of infection	
No	Sex ratio	hrate	RR	pIntro	ProbFree	2.5%	97.5%	ProbFree	2.5%	97.5%	90.0%	99.0%
**1**	ordinary	h1	1:2:6	5/0.1	0.617	0.602	0.631	0.893	0.881	0.904	6	11
2	ordinary*2	h1*2	1:2:6	5/0.1	0.695	0.676	0.712	0.982	0.977	0.985	3	6
3	1:3	h1	1:2:6	5/0.1	0.783	0.754	0.810	0.989	0.984	0.992	3	6
4	1:3	h0	1:2:6	5/0.1	0.779	0.753	0.805	0.992	0.989	0.995	3	5
5	1:5	h1	1:2:6	5/0.1	0.838	0.813	0.860	0.996	0.993	0.997	2	5
**6**	1:5	h0	1:2:6	5/0.1	0.835	0.811	0.857	0.997	0.996	0.998	2	4
7	1:10	h1	1:2:6	5/0.1	0.875	0.855	0.895	0.998	0.997	0.999	2	4
8	1:10	h0	1:2:6	5/0.1	0.872	0.850	0.892	0.999	0.998	0.999	2	4
9	1:20	h1	1:2:6	5/0.1	0.892	0.872	0.910	0.999	0.998	0.999	2	4
10	1:20	h0	1:2:6	5/0.1	0.890	0.869	0.909	0.999	0.999	0.999	2	3
11	ordinary	h1	1:1:1	5/0.1	0.595	0.583	0.606	0.870	0.858	0.882	6	NR
12	ordinary	h1	1:2:2	5/0.1	0.600	0.589	0.612	0.881	0.868	0.893	6	11
13	ordinary	h1	1:2:4	5/0.1	0.611	0.597	0.624	0.889	0.877	0.900	6	11
14	ordinary	h1	βp(1:2:6)	5/0.1	0.616	0.602	0.631	0.892	0.879	0.904	6	11

15	ordinary	h1	βp(1:1.5:4.5)	5/0.1	0.615	0.600	0.630	0.892	0.880	0.903	6	11
16	1:5	h0	1:1:1	5/0.1	0.701	0.683	0.719	0.957	0.948	0.964	4	8
17	1:5	h0	1:2:2	5/0.1	0.727	0.709	0.746	0.974	0.967	0.979	3	7
18	1:5	h0	1:2:4	5/0.1	0.798	0.777	0.820	0.993	0.990	0.995	3	5
19	1:5	h0	βp(1:2:6)	5/0.1	0.828	0.782	0.864	0.996	0.991	0.998	2	5
20	1:5	h0	βp(1:1.5:4.5)	5/0.1	0.827	0.786	0.862	0.996	0.992	0.998	2	5
21	ordinary	h1	1:2:6	5/5	0.592	0.577	0.605	0.804	0.788	0.820	10	NR
22	ordinary	h1	1:2:6	5/1	0.612	0.596	0.626	0.877	0.863	0.888	6	NR
23	ordinary	h1	1:2:6	5/0.5	0.615	0.600	0.630	0.886	0.873	0.898	6	NR
24	ordinary	h1	1:2:6	1/1	0.650	0.634	0.664	0.892	0.880	0.902	6	NR
25	ordinary	h1	1:2:6	1/0.1	0.655	0.640	0.669	0.908	0.897	0.918	5	11
26	1:5	h0	1:2:6	5/5	0.820	0.794	0.844	0.972	0.964	0.978	3	NR
27	1:5	h0	1:2:6	5/1	0.832	0.807	0.855	0.993	0.990	0.994	2	5
28	1:5	h0	1:2:6	5/0.5	0.834	0.810	0.858	0.995	0.993	0.996	2	5
29	1:5	h0	1:2:6	1/1	0.854	0.831	0.875	0.993	0.991	0.995	2	5
30	1:5	h0	1:2:6	1/0.1	0.856	0.833	0.875	0.997	0.996	0.998	2	4

Supplementary Table 4. An overview of animals sampled and demographic structure depending on harvest scenario in Supplementary Table 3 for Nordfjella zone 2, Norway. No = scenario in Supplementary Table 3. Prop. = proportion. Adf = adult females. Adm = adult males. Year denote sampling for 1 year (2018) or after 5 years (2022).

	**Number tested**		**Demographic structure**						
			Postharvest			Preharvest		Preharvest	
			prop. Adf	prop. Adm	prop. calves	Pop size		Pop size excl. calves	
No	After 2018	After 2022	2018	2018	2018	2018	2022	2018	2022
1	59	385	0.346	0.237	0.223	573	774	445	609
2	116	538	0.342	0.225	0.233	506	461	388	354
3	129	514	0.394	0.131	0.254	503	666	375	495
4	118	520	0.378	0.126	0.263	524	735	386	541
5	155	546	0.415	0.084	0.268	477	639	349	466
6	144	554	0.398	0.078	0.278	497	710	360	512
7	175	574	0.433	0.044	0.280	457	618	329	444
8	164	582	0.415	0.040	0.289	477	685	339	487
9	185	587	0.443	0.022	0.286	447	606	319	432
10	174	597	0.423	0.021	0.295	468	676	330	476

Supplementary Table 5. An overview of different harvest scenarios (strategy) for Hardangervidda, Norway. Sex ratios are ordinary (Supplmentary Table 2), increased harvest to twice the rate of ordinary (ordinary*2) or given with male:female ratios. hrate = harvest rate (h2 from Suppl. Table 2; h0 = no calves or yearling harvest). RR = relative risk (RR of yearlings, adult females and adult males relative to yearlings). Probability of infection introduction (pIntro) is baseline 1% first year and 0.1% thereafter. ProbFree = mean probability for freedom of infection. NR = not reached. A sensitivity analysis of pintro and RR is included for two harvest strategies (No1 and No8). A few scenarios run with stochastic RR were also included. Stochastic RR were assumed betapert distributed (βp) with minimum RR=1:1:2, maximum RR=1:2.5:6:5 and mode RR=1:2:6 or 1:1.5:4.5).

					After 1 year (2018)			After 5 years (2022)			Years to reach a given level of freedom of infection	
No	Sex ratio	hrate	RR	pIntro	ProbFree	2.5%	97.5%	ProbFree	2.5%	97.5%	90.0%	99.0%
**1**	ordinary	h2	1:2:6	1/0.1	0.695	0.687	0.703	0.935	0.930	0.941	4	10
2	ordinary*2	h2*2	1:2:6	1/0.1	0.776	0.767	0.785	0.984	0.981	0.986	3	6
3	ordinary*2	h2*2 (Tadf2800)	1:2:6	1/0.1	0.787	0.778	0.795	0.985	0.983	0.987	3	6
4	ordinary*2	h2*2 (Tadf2500)	1:2:6	1/0.1	0.803	0.794	0.811	0.988	0.986	0.989	2	6
5	1:3	h2	1:2:6	1/0.1	0.855	0.845	0.866	0.987	0.984	0.990	2	6
6	1:3	h0	1:2:6	1/0.1	0.851	0.840	0.862	0.992	0.991	0.994	2	5
7	1:5	h2	1:2:6	1/0.1	0.903	0.895	0.912	0.995	0.994	0.996	1	5
**8**	1:5	h0	1:2:6	1/0.1	0.900	0.892	0.908	0.997	0.997	0.998	1	4
9	1:10	h2	1:2:6	1/0.1	0.932	0.925	0.938	0.998	0.997	0.998	1	4
10	1:10	h0	1:2:6	1/0.1	0.930	0.923	0.936	0.999	0.998	0.999	1	4
11	1:20	h2	1:2:6	1/0.1	0.944	0.938	0.949	0.999	0.998	0.999	1	4
12	1:20	h0	1:2:6	1/0.1	0.942	0.936	0.948	0.999	0.999	0.999	1	3
13	ordinary	h2	1:1:1	1/0.1	0.663	0.652	0.672	0.912	0.904	0.920	5	11
14	ordinary	h2	1:2:2	1/0.1	0.663	0.651	0.673	0.912	0.904	0.921	5	11
15	ordinary	h2	1:2:4	1/0.1	0.684	0.675	0.693	0.928	0.922	0.935	5	10

16	ordinary	h2	βp(1:2:6)	1/0.1	0.693	0.678	0.705	0.934	0.923	0.942	5	10
17	ordinary	h2	βp(1:1.5:4.5)	1/0.1	0.692	0.680	0.704	0.933	0.924	0.942	5	10
18	1:5	h0	1:1:1	1/0.1	0.784	0.772	0.794	0.969	0.964	0.973	3	8
19	1:5	h0	1:2:2	1/0.1	0.801	0.790	0.812	0.980	0.977	0.983	3	7
20	1:5	h0	1:2:4	1/0.1	0.871	0.861	0.880	0.994	0.993	0.995	2	5
21	1:5	h0	βp(1:2:6)	1/0.1	0.896	0.862	0.919	0.997	0.993	0.998	2	4
22	1:5	h0	βp(1:1.5:4.5)	1/0.1	0.895	0.864	0.918	0.997	0.993	0.998	2	4
23	ordinary	h2	1:2:6	5/5	0.635	0.627	0.644	0.843	0.834	0.852	9	NR
24	ordinary	h2	1:2:6	5/1	0.656	0.647	0.665	0.911	0.905	0.917	5	NR
25	ordinary	h2	1:2:6	5/0.5	0.659	0.65	0.668	0.919	0.913	0.926	5	NR
26	ordinary	h2	1:2:6	5/0.1	0.661	0.652	0.669	0.926	0.919	0.932	5	10
27	ordinary	h2	1:2:6	1/1	0.691	0.683	0.699	0.921	0.915	0.927	5	NR
28	1:5	h0	1:2:6	5/5	0.875	0.865	0.885	0.970	0.967	0.974	2	NR
29	1:5	h0	1:2:6	5/1	0.885	0.875	0.894	0.992	0.991	0.993	2	5
30	1:5	h0	1:2:6	5/0.5	0.886	0.877	0.895	0.995	0.994	0.996	2	5
31	1:5	h0	1:2:6	5/0.1	0.887	0.878	0.896	0.997	0.996	0.998	2	4
32	1:5	h0	1:2:6	1/1	0.900	0.891	0.908	0.993	0.992	0.994	2	5

Supplementary Table 6. An overview of animals sampled and demographic structure depending on harvest scenario in Supplmentary Table 5 for Hardangervidda. Norway. No = scenario in Supplmentary Table 3. Prop. = proportion. Adf = adult females. Adm = adult males. Year denote sampling for 1 year (2018) or after 5 years (2022).

	**Number tested**		**Demographic structure**						
			Postharvest			Preharvest		Preharvest	
			prop. Adf	prop. Adm	prop. calves	Pop size		Pop size excl. calves	
No	After 2018	After 2022	2018	2018	2018	2018	2022	2018	2022
1	733	4988	0.410	0.272	0.183	7319	8170	5981	6342
2	1361	5903	0.463	0.240	0.173	6481	6019	5363	4578
3	1559	5993	0.446	0.247	0.178	6277	5644	5160	4293
4	1858	6142	0.418	0.260	0.187	5978	5081	4861	3874
5	1727	6125	0.474	0.158	0.211	6325	7276	4990	5441
6	1538	6421	0.446	0.149	0.231	6733	8638	5175	6305
7	2127	6630	0.506	0.101	0.226	5926	6886	4589	5049
8	1941	6885	0.474	0.095	0.245	6327	8282	4774	5939
9	2424	6991	0.533	0.053	0.237	5625	6605	4290	4758
10	2243	7241	0.498	0.050	0.258	6030	8001	4477	5647
11	2573	7167	0.548	0.027	0.244	5479	6459	4141	4616
12	2391	7427	0.510	0.026	0.264	5880	7872	4327	5507

Supplementary References

1. Nilsen, E. B. & Strand, O. Integrating data from several sources for increased insight into demographic processes: Simulation studies and proof of concept for hierarchical change in ratio models. *Plos One* **13**, e0194566(2018).
2. Mysterud, A., Coulson, T. & Stenseth, N. C. The role of males in the population dynamics of ungulates. *J Anim Ecol* **71**, 907-915 (2002).
3. Coulson, T., Mace, G. M., Hudson, E. & Possingham, H. The use and abuse of population viability analysis. *Trends Ecol Evol* **16**, 219-221 (2001).
4. Strand, O., Nilsen, E. B., Solberg, E. J. & Linnell, J. D. C. Can management regulate the population size of wild reindeer (*Rangifer tarandus*) through harvest? *Can J Zool* **90**, 163-171 (2012).
5. Viljugrein, H., Hopp, P., Benestad, S. L., et al. A method that accounts for differential detectability in mixed samples of long-term infections with applications to the case of Chronic Wasting Disease in cervids. *Methods Ecol Evol* **10**, 134-145 (2019).
6. European Food Safety Authority (EFSA). Scientific report of the European Food Safety Authority on the evaluation of rapid post mortem TSE tests intended for small ruminants. *EFSA Journal* **3**, 49r-n/a(2005).
7. Mitchell, G. B., Sigurdson, C. J., O'Rourke, K. I., et al. Experimental oral transmission of Chronic Wasting Disease to reindeer (*Rangifer tarandus tarandus*). *Plos One* **7**, e39055(2012).
8. Moore, S. J., Kunkle, R., Greenlee, M. H. W., et al. Horizontal transmission of Chronic Wasting Disease in reindeer. *Emerg Infect Dis* **22**, 2142(2016).

9. Fox, K. A., Jewell, J. E., Williams, E. S. & Miller, M. W. Patterns of PRP^{CWD} accumulation during the course of Chronic Wasting Disease infection in orally inoculated mule deer (*Odocoileus hemionus*). *J Gen Virol* **87**, 3451-3461 (2006).
10. Moore, S. J., Vrentas, C. E., Hwang, S., West Greenlee, M. H., Nicholson, E. M. & Greenlee, J. J. Pathologic and biochemical characterization of PrP^{Sc} from elk with *PRNP* polymorphisms at codon 132 after experimental infection with the chronic wasting disease agent. *BMC Veterinary Research* **14**, 80(2018).
11. Martin, P. A., Cameron, A. R. & Greiner, M. Demonstrating freedom from disease using multiple complex data sources. *Prev Vet Med* **79**, 71-97 (2007).
12. Mysterud, A., Madslien, K., Viljugrein, H., et al. The demographic pattern of infection with chronic wasting disease in reindeer at an early epidemic stage. *Ecosphere* **10**, e02931(2019).
13. Heisey, D. M., Osnas, E. E., Cross, P. C., Joly, D. O., Langenberg, J. A. & Miller, M. W. Linking process to pattern: estimating spatiotemporal dynamics of a wildlife epidemic from cross-sectional data. *Ecol Monogr* **80**, 221-240 (2010).
14. Samuel, M. D. & Storm, D. J. Chronic wasting disease in white-tailed deer: infection, mortality, and implications for heterogeneous transmission. *Ecol* **97**, 3195-3205 (2016).
15. Miller, M. W. & Conner, M. M. Epidemiology of Chronic Wasting Disease in free-ranging mule deer: Spatial, temporal, and demographic influences on observed prevalence patterns. *J Wildl Dis* **41**, 275-290 (2005).
16. Sargeant, G. A., Weber, D. C. & Roddy, D. E. Implications of chronic wasting disease, cougar predation, and reduced recruitment for elk management. *J Wildl Manage* **75**, 171-177 (2011).

17. Monello, R. J., Powers, J. G., Hobbs, N. T., Spraker, T. R., Watry, M. K. & Wild, M. A. Survival and population growth of a free-ranging elk population with a long history of exposure to Chronic Wasting Disease. *J Wildl Manage* **78**, 214-223 (2014).

18. Potapov, A., Merrill, E., Pybus, M. & Lewis, M. A. Empirical estimation of R_0 for unknown transmission functions: The case of Chronic Wasting Disease in Alberta. *Plos One* **10**, e0140024(2015).

19. Miller, M. W., Hobbs, N. T. & Tavener, S. J. Dynamics of prion disease transmission in mule deer. *Ecol Appl* **16**, 2208-2214 (2006).

20. Panzacchi, M., Van Moorter, B., Strand, O., et al. Predicting the continuum between corridors and barriers to animal movements using Step Selection Functions and Randomized Shortest Paths. *J Anim Ecol* **85**, 32-42 (2015).
