# Supplementary material for: Hunting strategies to increase detection of chronic wasting disease in cervids
Source: Nat Commun. 2020 Sep 1;11:4392. doi: 10.1038/s41467-020-18229-7 (PMC7463264; doi:10.1038/s41467-020-18229-7)
Supplement: Supplementary file 3 — Reporting Summary [file 41467_2020_18229_MOESM3_ESM.pdf]

nature research

Corresponding author(s): Atle Mysterud

Last updated by author(s): Jul 27, 2020

Reporting Summary

Nature Research wishes to improve the reproducibility of the work that we publish. This form provides structure for consistency and transparency in reporting. For further information on Nature Research policies, see Authors & Referees and the Editorial Policy Checklist .

Statistics

For all statistical analyses, confirm that the following items are present in the figure legend, table legend, main text, or Methods section.

n/a	Confirmed	
☐	☒	The exact sample size (*n*) for each experimental group/condition, given as a discrete number and unit of measurement
☐	☒	A statement on whether measurements were taken from distinct samples or whether the same sample was measured repeatedly
☒	☐	The statistical test(s) used AND whether they are one- or two-sided<br>*Only common tests should be described solely by name; describe more complex techniques in the Methods section.*
☐	☒	A description of all covariates tested
☐	☒	A description of any assumptions or corrections, such as tests of normality and adjustment for multiple comparisons
☐	☒	A full description of the statistical parameters including central tendency (e.g. means) or other basic estimates (e.g. regression coefficient) AND variation (e.g. standard deviation) or associated estimates of uncertainty (e.g. confidence intervals)
☒	☐	For null hypothesis testing, the test statistic (e.g. *F*, *t*, *r*) with confidence intervals, effect sizes, degrees of freedom and *P* value noted<br>*Give P values as exact values whenever suitable.*
☐	☒	For Bayesian analysis, information on the choice of priors and Markov chain Monte Carlo settings
☐	☒	For hierarchical and complex designs, identification of the appropriate level for tests and full reporting of outcomes
☐	☒	Estimates of effect sizes (e.g. Cohen's *d*, Pearson's *r*), indicating how they were calculated

Our web collection on statistics for biologists contains articles on many of the points above.

Software and code

Policy information about availability of computer code

Data collection	Data is the sum of all data recorded (computerized) for number of animals harvested, number of animals tested for CWD as well as total counts of animals from population surveys, and no code was used.
Data analysis	R version 3.6.2 (https://github.com/NorwegianVeterinaryInstitute/Hunting_wildlife_for_disease_detection)

For manuscripts utilizing custom algorithms or software that are central to the research but not yet described in published literature, software must be made available to editors/reviewers. We strongly encourage code deposition in a community repository (e.g. GitHub). See the Nature Research guidelines for submitting code & software for further information.

Data

Policy information about availability of data

All manuscripts must include a data availability statement. This statement should provide the following information, where applicable:

- Accession codes, unique identifiers, or web links for publicly available datasets
- A list of figures that have associated raw data
- A description of any restrictions on data availability

All data and codes is available on github (https://github.com/NorwegianVeterinaryInstitute/Hunting_wildlife_for_disease_detection)

Field-specific reporting

Please select the one below that is the best fit for your research. If you are not sure, read the appropriate sections before making your selection.

☐ Life sciences ☐ Behavioural & social sciences ☒ Ecological, evolutionary & environmental sciences

Ecological, evolutionary & environmental sciences study design

All studies must disclose on these points even when the disclosure is negative.

Study description	Main parts: population data used in a population estimation model. Output from population estimation model used in a population simulation model. Output from the population simulation model feeded into a disease detection model - where tested samples for CWD was fed into. Output was an optimal harvest strategy to establish freedom-of-disease.
Research sample	Two main data sources all from reindeer Rangifer tarandus: (1) Four population surveys from each of two populations. (2) testing for CWD for all harvested reindeer (1 yr or older) in both populations. The rationale for choice of populations was their closeness to the population in Nordfjella zone 1 previously infected with chronic wasting disease.
Sampling strategy	A part of the paper is simulations guiding harvest quotas - hence, indirectly determining sampling strategy
Data collection	Two sources: (1) population data derived from four surveys (calving surveys and minimum winter counts from airplane; demographic structure count from ground; hunter data from reported harvest to local managers) and (2) samples tested for CWD and being registered in the internal data base at the Norwegian Veterinary Institute. The population surveys are performed by Norwegian Institute for Nature Research in collaboration with local reindeer management authorities. The actual people involved has changed over time in each on the institutions. The number of hunted animals is registered and reported by Statistics Norway on behalf of the Norwegian Environment Agency.
Timing and spatial scale	Spatial scale were two demarcated populations (Hardangervidda and Nordfjella zone 2). Temporal scale - annual population data from as far back as we could retrieve (Hardangervidda: 2000-2018 and Nordfjella zone 2: 2015-2018). Disease surveillance data - all data from after CWD was discovered and testing initiated (annual 2016-2019 in both populations: one extra survey winter 2018 in Nordfjella zone 2)
Data exclusions	All data used
Reproducibility	Given our data, all codes will be published to reproduce our results
Randomization	No randomization, all incoming samples were tested.
Blinding	Blinding is not possible

Did the study involve field work? ☐ Yes ☒ No

Reporting for specific materials, systems and methods

We require information from authors about some types of materials, experimental systems and methods used in many studies. Here, indicate whether each material, system or method listed is relevant to your study. If you are not sure if a list item applies to your research, read the appropriate section before selecting a response.

Materials & experimental systems

n/a	Involved in the study
☒	☐ Antibodies
☒	☐ Eukaryotic cell lines
☒	☐ Palaeontology
☐	☒ Animals and other organisms
☒	☐ Human research participants
☒	☐ Clinical data

Methods

n/a	Involved in the study
☒	☐ ChIP-seq
☒	☐ Flow cytometry
☒	☐ MRI-based neuroimaging

Animals and other organisms

Policy information about studies involving animals; ARRIVE guidelines recommended for reporting animal research

Laboratory animals	NA
Wild animals	Data comes from population surveys of reindeer and based on testing ordinary harvest data from hunters for disease (ie. citizen science data)
Field-collected samples	NA
Ethics oversight	Not required - as data derive from ordinary harvesting

Note that full information on the approval of the study protocol must also be provided in the manuscript.
